# Prenatal stem cell therapy for inherited diseases: Past, present, and future treatment strategies

**DOI:** 10.1002/sctm.19-0107

**Published:** 2019-10-24

**Authors:** Åsa Ekblad‐Nordberg, Lilian Walther‐Jallow, Magnus Westgren, Cecilia Götherström

**Affiliations:** ^1^ Department of Clinical Science, Intervention and Technology, Division of Obstetrics and Gynecology Karolinska Institutet Stockholm Sweden

**Keywords:** cell therapy, inherited diseases, prenatal, stem cell, treatment strategies

## Abstract

Imagine the profits in quality of life that can be made by treating inherited diseases early in life, maybe even before birth! Immense cost savings can also be made by treating diseases promptly. Hence, prenatal stem cell therapy holds great promise for developing new and early‐stage treatment strategies for several diseases. Successful prenatal stem cell therapy would represent a major step forward in the management of patients with hematological, metabolic, or immunological disorders. However, treatment before birth has several limitations, including ethical issues. In this review, we summarize the past, the present, and the future of prenatal stem cell therapy, which includes an overview of different stem cell types, preclinical studies, and clinical attempts treating various diseases. We also discuss the current challenges and future strategies for prenatal stem cell therapy and also new approaches, which may lead to advancement in the management of patients with severe incurable diseases.

## Graphical abstract

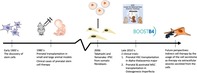

**Significance statement**

This review summarizes the past, the present progress, and the future potential of prenatal stem cell therapy. Recent and previous studies are discussed, focusing on both preclinical and clinical data, highlighting both the drawbacks and the novel findings leading to the progress of prenatal stem cell therapies into the clinic.

## 1 INTRODUCTION TO STEM CELL THERAPY

**Figure 1:**
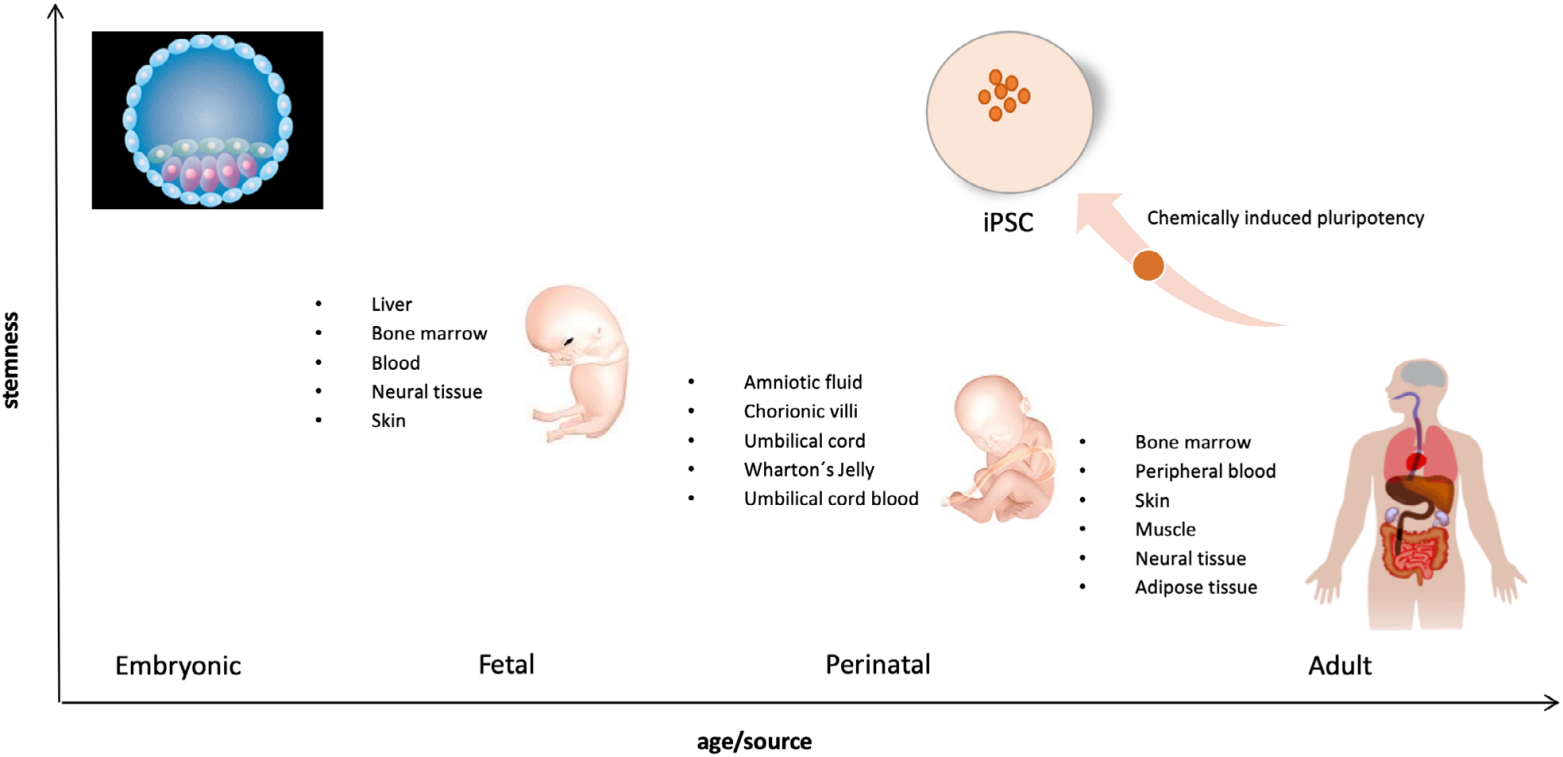
Different sources for isolation of stem cells and their shift in stemness during aging. Stem cells can be isolated from many sources during development. In general, the earlier in development, that is, the younger the donor tissue is, the more potential and stemness the isolated stem cells exhibit. Induced pluripotent stem cells (iPS) are the exception to this concept. Illustrations from https://commons.wikimedia.org

Cell therapy is by definition the administration of living cells to patients to replace or repair damaged or dysfunctional organs or tissue. The cells can originate from the patients themselves (autologous) or from human leukocyte antigen (HLA) matched or mis‐matched donors (allogeneic). The cells used for therapy can have different potentiality (Table 1 and Figure 1), and can be unstimulated or in vitro differentiated.^1^, ^2^ The cells can be administered intravenously or directly applied into the damaged organ or tissue. The main mechanism of action for stem cell therapies is donor cell engraftment and subsequent differentiation and replacement of damaged tissue or secondly, and more recently investigated, via trophic effects by secretion of soluble factors such as cytokines, growth factors, or chemokines, by the donor cell.

**Table 1:** Different stem cell populations, their sources. and respective clinical potential and usability

Cell populations	Sources	Clinical potential and usability
Adipose‐derived stem cells (ADSC)	White adipose tissue	Adipose tissue is abundant in the human body and large amount of ADSC can easily be isolated with minimal donor site morbidity. The vast number of published preclinical studies of the ADSC reveals among other things the pro‐angiogenic properties, and that the cells promote wound healing and tissue regeneration.^77^ ADSC displays mesenchymal features but are more abundant and possess greater in vitro anti‐inflammatory effects than bone marrow mesenchymal stem cell (BM‐MSC).^77^ These preclinical studies also provided evidence on the safety and efficacy of ADSC and several clinical trials regarding, for example, immune, orthopedic or soft tissue defects are currently ongoing.^77^, ^78^
Cardiac progenitor cells	Heart tissue	Fetal cardiac progenitor cells drive the growth of the developing heart through proliferation and possess regenerative properties. After birth both the proliferative and regenerative properties are diminished and the cells may exit the cell cycle. The existence of adult cardiac progenitor cells is controversial. Scientists discovering proliferative and thereby regenerative cells have most often detected DNA synthesis in polynucleated cardiomyocytes, which did not re‐enter the cell cycle.^79^ Postnatal c‐KIT+ cardiac progenitor cells (CPC) have been reported to give rise to cardiomyocytes, smooth muscle cells and endothelial cells, and autologous c‐KIT+ CPC has entered a phase I study while other studies suggest that 90%‐100% of all of the cardiac c‐KIT+ cells are actually mast cells.^80^ For cell therapeutic purpose, cardiac progenitor cells seem unsuitable and other stem cells are being investigated, such as lineage‐specified cardiopoietic MSC or stem cells differentiated from embryonic stem cell (ESC) or iPSC from heart fibroblasts.^81^
Endothelial progenitor cells (EPC)	Peripheral blood, spleen, vessel walls, and bone marrow	EPC are matured from basal cells, and home to sites of vascular injury to restore vascular homeostasis and promotes neovascularization. After intracardiac injection of EPC in animal models of ischemia, blood perfusion was improved and intravenously administered autologous EPC increased cardiac function and reduced ventricular scarring after induced myocardial infarction, indicating promising therapeutic potential of the EPC. However, clinical studies with EPC as cellular therapy for ischemia could indeed present improved pathological features, although little or no clinical benefit could be observed. Therefore, potential clinical applications of EPC as cell therapy should await further safety, feasibility and efficacy studies before moving further toward the clinic.^82^
Hepatic stem cells	Liver tissue	Hepatic stem cells have shown promising results as cell therapy for liver diseases when distributed via the portal vein. The cells homed and integrated into the lobes with cumulative decreased disease severity index (Mayo's Model for End‐Stage Liver Disease) after stem cell distribution. The suggested source of stem cells is fetal tissues, as pediatric and adult livers are preferred as subjects for organ transplantation due to the constant lack of donor organs.^83^
iPSC	Somatic cells	iPSC can in theory replace any pluri‐ or multipotent stem cell population for cell therapy and enables development of personalized treatment based on an autologous cell source. The challenge is to develop a robust differentiation method producing pure and uniform differentiated populations and to apply safety requirement to avoid teratoma formations.^84^ Another concern is that the iPSC maintain the methylation state or epigenetic memory associated with their somatic cell‐of‐origin state which may influence their potential to differentiate into the cell type of interest.^85^
Neural stem cells (NSC)	Central nervous system	NSC are rare populations in the brain and the access of human brain tissue is very limited. Previous isolations of viable NSC from human postmortem brain tissue showed reduced number of NSC with reduced proliferative capacity in the adult brains compared to prenatal brain tissue.^86^ In contrast, NSC are more abundant in the developing early fetal brain and have been used in several clinical trials for treating neurodegenerative diseases.^87^ To obtain a sufficient number of cells, multiple donors are required and hence with limited access to suitable donors and due to ethical considerations, scientists are considering using ESC or iPSC as an alternative source for replacement therapies for neurodegenerative diseases.^88^ Recently a FDA‐approved stem cell line (NSI‐566) derived from first trimester fetal spinal cord was established and the use has led to promising results in phase I and II clinical studies of neurodegenerative diseases and spinal cord injuries.^89^
Satellite cells	Skeletal muscle tissue	Satellite cells, the muscle stem cells, possess self‐renewal capacity and lineage commitment toward myogenic tissue, making them attractive for therapeutic purposes. The need for harvesting large amount of tissue to obtain sufficient material for muscle stem cell therapy however, made this approach unachievable. The successful collection of viable satellite cells from postmortem tissue may circumvent that obstacle.^90^

Included in the table are selected stem cell types with a possible relevance to stem cell therapy. MSC and hematopoietic stem cell (HSC) are not included in the table since they are discussed extensively in the article.

## 2 POTENTIAL FOR PRENATAL STEM CELL THERAPIES

Many congenital diseases manifest early and thus prenatal diagnosis is often possible as early as 10‐12 weeks of gestation. Our knowledge about the natural history of prenatally diagnosed disorders is often limited but at least in some cases it is known that damage continues to develop as gestation advances. Hence, there is a rationale for treatment to be initiated as early as possible, thereby ameliorating pathology or even preventing it from occurring. An improved situation for the fetus at birth might hypothetically benefit the transition from fetal to neonatal life and thus improve perinatal survival. Even though a single prenatal infusion may not be clinically sufficient for permanent improvement, a prenatal treatment approach is still justifiable since the immunological naïveté of the fetus may allow for the development of immune tolerance toward the donor cells, rendering postnatal booster treatments more efficient. Benefits of initiation of treatment of for example severe types of osteogenesis imperfecta (OI) during fetal life compared to postnatally include reduced risk of additional fractures and bowing of long bones before birth, improvement in intrauterine growth, and earlier engraftment and hence increased effect of the transplanted cells. Lastly, during fetal life there are better physiological conditions for systemic distribution of the infused cells because of the presence of circulatory shunts. When the donor cells are administered into the umbilical vein of the fetus, the cells will bypass the pulmonary circulation via two fetal shunts, the ductus arteriosus and the foramen ovale. This ensures that the donor cells go directly into the systemic circulation and widely into the peripheral organs. Administration of cells after birth is performed into a peripheral vein, with many cells becoming trapped in the microcirculation of the lungs,^3^, ^4^, ^5^, ^6^, ^7^ resulting in fewer cells reaching the systemic circulation. Other benefits of prenatal treatment include a better psychosocial situation for the mother and father from the birth of a child who has already been treated, and possibly an improved parent and family quality of life. Some of these benefits might also hold true for other types of inherited congenital disorders.

The use of high‐resolution ultrasound has paved the way for early gestational diagnosis and cellular transplants and the procedure can be considered as safe, both for the fetus and the pregnant woman. The prenatal infusion procedure is comparable to intrauterine blood transfusions, which are routinely performed in expert fetal therapy centers and where procedure safety (currently a procedure complication rate of 1.2% and a procedure loss rate of 0.6%) has improved significantly during the last 20 years due to improved transfusion techniques.^8^

## 3 STEM CELLS

Ever since Alexander Maksimov proposed the existence of stem cells in the beginning of the 1900s,^9^ researchers have been searching for and identified stem cells from human tissue sources. There is a hierarchical capacity of self‐renewal and differentiation ability of human stem cells ranging from the immature totipotent fertilized egg capable of forming all embryonic and extraembryonic tissues, the pluripotent embryonic cells that forms all cells in the developing fetus, the multipotent lineage restricted tissue residing stem cells, the oligopotent more restricted progenitor cells, to the mature unipotent precursor cells which only forms one cell type.^10^ In addition to conventional stem cells, the work by Takahashi and Yamanaka demonstrated that induced expression of four embryonic transcription factors could transform terminally differentiated somatic cells into induced pluripotent stem cells (iPSC, Table 1 and Figure 1).^11^ An increasing number of distinctive stem cell populations have been identified and isolated from almost all tissues in the human body. These cells have different potential, which includes their respective “stemness” potential (Figure 1). The proposed clinical applications increases as new categories of severe clinical conditions becomes prospective candidates for stem cell therapies. Currently there are approximately 1000 registered clinical trials with human stem cell therapies (http://clinicaltrials.gov, September 2019). The different types of stem cells relevant to stem cell therapy, as of now and in the nearest future, are described in detail below and in Table 1 together with their source and possible clinical potential.

### 3.1 Totipotent stem cells

The first four cells in the embryo are totipotent and hence possess great potential, but are not eligible candidates for stem cell therapies and will not be further discussed here.

### 3.2 Pluripotent stem cells

Embryonic stem cells (ESCs) are derived from the inner cell mass of the early embryo. Both the ESC and the iPSC in the immature pluripotent state form teratomas if transplanted and must be differentiated to a more mature and safe state before transplantation (Table 1 and Figure 1).

### 3.3 Multipotent stem cells

Multipotent stem cells have good self‐renewing potential but not to the same extent as pluripotent cells. Importantly, they do not form teratomas and do not necessarily need to be differentiated before transplantation.

Hematopoietic stem cells (HSC) derived from bone marrow (BM), mobilized into peripheral blood, umbilical cord blood (UCB), or fetal liver was the first stem cell type to be discovered, and is the most extensively investigated stem cell population, both experimentally and clinically. During the last 30 years, allogeneic HSC transplantation has become the major therapeutical approach for treatment of hematopoietic disorders such as leukemias, disorders of the immune system, and metabolic disorders and more than 40 000 transplantations using HSC are performed annually in Europe (http://www.ebmt.org).^12^, ^13^ After transplantation, HSC migrate to the BM, where they self‐renew and reconstitute the defect hematopoietic system.

BM or peripheral blood from HLA matched donors are the most frequently used sources for HSC transplantation. These sources rely on the availability of family donors and worldwide registries with volunteer donors. UCB, on the other hand, is readily available from cord blood banks and complete HLA‐matching is not needed.^13^ However, one UCB unit may not be sufficient for an adult recipient, and infusion of donor lymphocytes is not possible due to the one single collection of a restricted volume of UCB.

Another promising multipotent cell is the mesenchymal stem or stromal cell (MSC). MSC can differentiate into mesodermal lineages, which is favorable in tissue reconstruction. MSC can be isolated from various tissues such as adipose, placenta (PL), umbilical cord (UC), UCB, amniotic fluid (AF), pre‐ and postnatal skin, and fetal liver, but the most common source is still adult BM.^14^ MSC are minimally immunogenic and do not induce an immune response at transplantation,^15^ hence an off‐the‐shelf transplantation approach is possible using allogenic nonmatched MSC. After transplantation, MSC home to sites of injury and inflammation and secrete trophic factors that enhances angiogenesis and endogenous tissue repair.^16^ MSC have a good safety profile and there are no reports on transformation after expansion. These properties make MSC clinically interesting and have been used for treatment for, for example, hematological pathologies, cardiovascular, neurological, bone, cartilage, autoimmune, and inflammatory diseases and for support of solid organ transplants.^17^ Up to date, thousands of patients have received treatment, mostly with adult BM‐MSC, with few adverse events reported.^18^

MSC isolated from fetal tissues have more potential in comparison to adult sources and have advantages for cell therapy compared to adult MSC, as discussed in the section below. The main issues with fetal tissue as a cellular source is the ethical considerations and the scarce accessibility of tissue. Another source is perinatal tissues, which are easily accessible high‐abundant sources for isolation of MSC and do not pose any major ethical concerns. MSC isolated from umbilical cord (UC‐MSC) and the fetal part of the placenta (PL‐MSC) possess a MSC phenotype equal to adult BM, but UC‐MSC have higher colony forming capacity and differentiation potential, whereas PL‐MSC display reduced colony forming capacity and equal or less differentiation potential when compared to adult BM‐MSC.^19^ AF‐derived MSC (AF‐MSC) can be isolated from mid‐ to late‐gestational AF samples with low risk for the woman and fetus.^20^ AF cells is an attractive source for autologous cell therapy with low ethical considerations coupled to the collection, and many preclinical cell therapy studies have presented promising results for, for example, cardiovascular, neuronal, and respiratory injury or damage.^21^ The drawback with AF is the heterogeneity of the cell content and the high donor variation.^22^

The skin is the body's largest organ and skin‐residing stem cells are rather accessible. MSC has been successfully isolated from both pre‐ and postnatal human dermis and subcutaneous tissue.^23^, ^24^ Fetal subcutaneous MSC‐like cells show high proliferative capacity which is favorable for the cell yield, but still within the Hayflick limit indicating stable, non‐embryonic cell phenotypes.^24^, ^25^ Adult dermal MSC promotes wound healing and modulates immune responses in mice,^26^ which show that these cells have similar characteristics as adult BM‐MSC.

Other clinical relevant tissue specific stem and progenitor cells are presented in Table 1.

### 3.4 What are the advantages of using fetal cells in prenatal cell therapy?

Fetal stem cells possess an intermediate phenotype between embryonic and adult cells that makes them ideal candidates for regenerative medicine since they have more potential but are not tumerogenic. Also, transplanting fetal cells to a fetus may produce higher engraftment than transplanting adult cells to a fetus. One preclinical study indicate that the recipient microenvironment may regulate the engraftment efficiency of a given stem cell source (read donor age).^27^ Prenatal transplantation of fetal liver cells (the blood forming organ during fetal life) had a 10‐fold competitive engraftment advantage relative to adult BM HSC in allogeneic fetal severe combined immunodeficient (SCID) recipients compared with adult recipients. In contrast, adult BM HSC engrafted slightly better than fetal liver cells in allogeneic adult SCID transplant recipients. Fetal liver cells repopulated 8.2 times better than adult BM HSC in fetal recipients, but only 0.8 times as well in adult recipients. Therefore, a fetal‐to‐fetal transplantation approach may be preferred in prenatal cell therapy.

Fetal‐derived MSC are similar to adult BM‐derived MSC displaying the same phenotype and low immunogenicity,^28^, ^29^ but have a number of potential advantages over adult MSC relevant to their potential use in cell therapy. Fetal MSC are found at a higher frequency, have a greater colony‐forming capacity, and have longer telomeres and a superior proliferative potential compared to MSC from adult sources.^29^, ^30^, ^31^, ^32^ Furthermore, fetal MSC differentiate more readily into bone, muscle, and oligodendrocytes compared to adult MSC.^31^, ^33^, ^34^, ^35^ In direct comparison of fetal MSC (liver, blood, and BM cells from the first trimester) and adult BM‐MSC, fetal MSC had higher levels of 16 osteogenic genes under basal conditions (noninduced to bone) than adult BM‐MSC.^33^ Upon osteogenic differentiation, fetal MSC displayed a more robust osteogenic gene expression and induced more calcium production in vitro and reached higher levels of osteogenic gene upregulation in vivo and in vitro than adult BM‐MSC.^33^ In another direct comparison, fetal first‐trimester BM‐MSC were compared to MSC derived from the term UC, adult adipose tissue, and adult BM. It was shown that all MSC had equivalent immunophenotype but that the fetal MSC exhibited the greatest osteogenic capacity, as assessed by von Kossa staining, alkaline phosphatase activity, calcium deposition, calcium visualized on microcomputed tomography and scanning electron microscopy, and osteogenic gene induction.^31^ These characteristics of fetal MSC makes them an interesting source for treatment of bone‐related diseases.

## 4 PRECLINICAL STUDIES AND CLINICAL TRIALS IN FETAL THERAPY

### 4.1 Past discoveries

The result of allogenic prenatal transplantations in experimental small and large animal models generated interest early on since engraftment was achieved and hence the potential for clinical therapy was understood. Mouse studies showed that it was possible to achieve derivation of myeloid and lymphoid lineages from HSC and tolerance in nondefective fetal mice after prenatal transplantation.^36^, ^37^, ^38^ Several studies investigated prenatal allogenic transplantation of HSC to sheep and nonhuman primates and showed long‐term engraftment of donor cells with multilineage differentiation (erythroid, myeloid, and lymphoid).^39^, ^40^, ^41^, ^42^ Donor cell engraftment was achieved without the use of cytoablation or immunosuppression and without the development of GvHD. Taken together, stem cell transplantation in larger animal models demonstrated tolerance and engraftment, paving the way for prenatal transplantation in humans.

This was followed by several attempts around the world to clinically treat various hematologic disorders with prenatal transplantation. The targeted disorders were were immundeficiencies, alpha‐ and beta‐thalassemia, and some inherited metabolic disorders. The first successful prenatal transplantation in humans was performed for bare lymphocyte syndrome, a rare immunodeficiency disorder, and was reported by Touraine et al.^43^ Following this case, transplantation of fetuses with other immunological disorders such as SCID was carried out in a number of centers, but with varying grade of success.^44^, ^45^, ^46^, ^47^ In these cases, fetal liver, paternal BM, or maternal BM‐derived CD34+ cells were transplanted between 16 and 26 weeks of gestation and resulted in engraftment of donor cells at birth and T‐cell recovery, but often failing B‐cell reconstitution. The cases vary in regard to mode of transplantation, transplant source, and gestational age and, therefore, it is cumbersome to make any general conclusions with regard to efficiency of the treatment in these cases. One could conclude that although these results to a certain extent looked promising, the method did not move into clinical practice, and the prevailing treatment for these children is postnatal BM transplantation.

To date, prenatal transplantation has been performed on 46 human patients for 14 different genetic disorders, including hemoglobinopathies, chronic granulomatous disease, Chediak‐Higashi syndrome, and inborn errors of metabolism^48^, ^49^, ^50^, ^51^, ^52^, ^53^ (reviewed in Reference 50). These studies have collectively provided evidence that the early human fetus can be accessed multiple times with a low procedure‐related risk, assuming that a minimally invasive, ultrasound‐guided approach is used.^52^, ^53^, ^54^, ^55^, ^56^, ^57^ With the notable exception of patients with SCID, the clinical experience thus far with prenatal transplantation using HSC has been largely disappointing. SCID is a unique disorder that provides a survival and proliferative advantage for donor T‐cells, and the engraftment achieved in these patients has only been documented to reconstitute the T‐cell lineage (split chimerism),^55^ just as was observed in the early experimental work in mice performed by Blazar et al.^38^ The results of the 46 clinical prenatal transplantation cases performed to date have clearly demonstrated that prenatal transplantation, using currently used methods, is not able to establish clinically relevant/therapeutic levels of engraftment in recipients whose hematopoietic system exhibits a normal level of competitiveness. Thus, current development in this field focus on finding new strategies for the donor cells to achieve a competitive advantage over the endogenous stem cells of the fetal recipient. For example, a recent study showed that fetal injection of antibodies against the c‐Kit receptor and CD47 effectively depleted host HSC in immunocompetent mice, which led to improved long term donor cell engraftment after neonatal HSC transplantation at postnatal day 0.^58^ This may be applied to prenatal transplantation. Also, better understanding of the fetal‐maternal interactions may improve the outcome after prenatal transplantation. It has been shown that maternal alloantibodies limit the long‐term engraftment following prenatal HSC transplantation in mice.^59^, ^60^ Maternal alloantibodies are transferred to pups through breast milk, which induce a postnatal adaptive immune response by the recipient, which in turn result in the ablation of engraftment after postnatal transplantation of HSC. When the recipients were fostered by surrogate mothers, they all maintained long‐term engraftment.

### 4.2 Present applications

Below, examples of preclinical investigations, case studies as well as clinical trials where prenatal cell therapy is an option for treatment of two selected diseases (Alpha‐Thalassemia Major and Osteogenesis Imperfecta) are presented.

#### 4.2.1 Alpha‐thalassemia major

As described above, most effort has been put into research on prenatal transplantation of HSC for their use in hematological derived diseases, but with little therapeutic success. The first systematic clinical investigation of prenatal HSC transplantation is currently performed for treatment of alpha‐thalassemia major and is led by Professor Tippi C. MacKenzie at the University of California, San Francisco. Alpha‐thalassemia major is almost uniformly fatal in utero without intervention. These fetuses have little circulating hemoglobin, and the hemoglobin that is present is all tetrameric γ chains, which are poor carriers of oxygen. The fetuses develop severe anemia, which result in hydrops fetalis that includes heart failure. The phase I trial aims to demonstrate the safety, feasibility and efficacy of administrating one dose of CD34+ enriched HSC to fetuses affected with alpha‐thalassemia major. The HSC are derived from the mother's BM and are infused into the umbilical vein between gestational week 18 to 25 just before one of the clinically indicated blood transfusions in utero. The rationale for using HSC from the mother is that the fetus will tolerate the mother's cells during pregnancy and the fetus will therefore not require any immune suppression. The trial will include 10 participants and is currently recruiting (http://clinicaltrials.gov ID: NCT02986698).

#### 4.2.2 Osteogenesis imperfecta

OI, also known as brittle bone diseases, is a group of genetic disorders that mainly affect the bones. Diagnosis of OI is usually made mid‐pregnancy using ultrasound, where the characteristic shortened long bones and fractures are already present and detectable. An analysis of the DNA confirms the diagnosis. The severity ranges from mild over severe to lethal. Individuals with OI suffer from multiple fractures, sometimes hundreds in a lifetime, osteopenia, short height, scoliosis, and chronic pain. The underlying mechanism is most commonly (>90%) a problem with connective tissue due to a lack of type I collagen that is caused by a mutation in the COL1A1 or COL1A2 genes. There is no cure or efficient treatment of OI.

A number of preclinical studies demonstrate that in mouse models of OI, MSC transplanted prenatally or in early neonatal life, equivalent to a late pregnancy human fetus, results in widespread distribution and engraftment of donor cells, and homing to the bones where the cells contribute to the osteoprogenitor population (donor cell engraftment between 0.3% and 28%), with improvement in collagen content, bone mineralization, and new bone formation.^61^, ^62^, ^63^, ^64^, ^65^, ^66^ In addition, one study showed that prenatal BM transplantation eliminated the perinatal lethality of the OI mice.^61^

One clinical trial has investigated postnatal transplantation of adult BM‐derived MSC in six children with severe types of OI and showed low‐level MSC engraftment in bone (<1%), reduced fracture frequency and growth stimulation (from a median of 20% increase 6 months before MSC transplantation to 60%‐94% increase 6 months after MSC transplantation) in five of six children.^67^ The effect was transient and lasted for approximately 6 months. Case studies of prenatal MSC transplantation for the treatment of OI also show clinical potential. We have reported the clinical course of two patients with OI types III and IV who received human fetal MSC transplantation prenatally with subsequent postnatal boosting with same‐donor MSC, resulting in low‐level engraftment in bone (0.003%‐16.6%) and improved linear growth, mobility, and fracture incidence, particularly when compared to individuals with an identical OI‐causing mutation.^68^ Neither patient demonstrated alloreactivity toward the donor MSC or manifested any evidence of toxicities after transplantation. Two more children suffering from severe OI have been treated prenatally or postnatally with fetal MSC with good results.^69^ These findings suggest that prenatal and postnatal transplantation of allogeneic fetal MSC in OI appears safe and is of likely clinical benefit and that retransplantation with same‐donor cells is feasible.

Postnatal or a combination of prenatal and postnatal treatment of severe types of OI with fetal MSC will be investigated in the phase I/II safety and efficacy Boost Brittle Bones Before Birth (BOOSTB4) trial (http://clinicaltrials.gov ID: NCT03706482), which is supported by the European Union's Horizon 2020 research and innovation program under grant agreement 681045 and the Swedish Research Council. Regulatory and ethical approval has been granted in Sweden and the United Kingdom, and recruitment has commenced.

### 4.3 Future perspectives

The therapeutic effect of MSC is not only related to their ability to engraft and differentiate into, for example, osteoblasts, chondrocytes, stroma, and adipocytes, and hence aid in regenerating different types of damaged tissue. Emerging data also indicate that soluble factors released by MSC are responsible for the beneficial outcomes; the so‐called paracrine effect, that triggers the body's own regenerative machinery. Part of this effect is assigned to extracellular vesicles (EVs, also called exosomes); nanometer‐sized, lipid membrane‐enclosed vesicles that are secreted by most cells and contain macromolecular material of the source cell including lipids, proteins, and various nucleic acid species. EVs act as important mediators of intercellular communication that influence both physiological and pathological conditions.^70^, ^71^ Owing to their ability to transfer bioactive components and surmount biological barriers, EVs are increasingly being explored as therapeutics, both for their innate abilities in tissue regeneration and immune modulation as potential alternatives to stem cell therapy.

A recent concise review found that there are over 200 preclinical studies of EV‐based therapies in a number of different animal models.^72^ For example, a recent paper showed that EVs derived from human AF‐derived stem cells could enhance cardiac function in mice.^73^ Also, MSC derived from the human UC have been shown to reduce neuroinflammation and induce neuroregeneration in perinatal brain injury in 3‐day old Wistar rat brains.^74^ A follow‐up study showed similar results with EVs derived from the same cell type, thus implying EVs as a promising therapy to prevent and treat perinatal brain injury.^75^

The possibility of harnessing and/or predict paracrine factors, including the secretome of EVs would be of great benefit to the field of stem cell therapy as a whole, providing possibilities to steer regeneration of tissues and organs in the desired direction while possibly avoiding graft rejection. A combination of cell transplantation including cell‐derived paracrine factors such as EVs, material science and bioengineering to construct new functional organs would be an attractive alternative instead of having to rely on organ and tissue transplantation and would open up endless possibilities for the future of regenerative medicine. Approaches to make use of engineered EVs as technology platforms in therapeutics have been evaluated. EVs are easy to manufacture and to bioengineer with multiple factors while maintaining their biostability. Furthermore, since they are small and acellular, they might avoid entrapment in different organs, for example, the lungs and may potentially also be able to cross biological barriers.^76^

## 5 ETHICAL CONSIDERATIONS

One cannot underestimate neither the need for a well‐controlled production of stem cells to be used as a medicinal product, nor the ethical considerations surrounding prenatal stem cell therapy and stem cell treatments. First, the source of the stem cells need to be well controlled so that the material is obtained with respect of ethical principles toward the donors. Furthermore, the cells need to be isolated, expanded, and quality controlled according to high‐quality standards under Good Manufacturing Practice conditions, as would any Advanced Therapy Medicinal Product. Clinically, prenatal diagnosis must be accurate and reliable and must be communicated in such a way that the parents are able to make a well informed and well‐founded decision of whether to agree to a prenatal stem cell therapy or not. Even though the outcome of a prenatal cell therapy may be very uncertain, the availability of a treatment before birth may affect the parents decision to continue or terminate the pregnancy. Lastly, one must keep in mind that apart from the fetus being treated, also the woman carrying the fetus might be affected by the treatment, further widening the need for a relevant ethical discussion.

## 6 CONCLUSION AND LOOKOUT INTO THE FUTURE

Prenatal stem cell therapy, with all their in‐built possibilities, can be a powerful tool to cure the incurable, already before birth. However, the limited clinical experience to date, often on heterogeneous case studies, means that it is not possible to be conclusive, and systematic clinical trials are vital to evaluate if a prenatal cell therapy approach is a realistic therapeutic alternative in specific diseases. In summary, establishment of prenatal stem cell therapy as a part of fetal therapy looks promising but bears several ethical and medical issues that must be addressed.

## CONFLICT OF INTEREST

The authors indicated no potential conflicts of interest.

## AUTHOR CONTRIBUTIONS

The authors have contributed equally to this review.

## DATA AVAILABILITY STATEMENT

Data sharing is not applicable to this article as no new data were created or analyzed in this study.
